# Cardiovascular outcomes associated with SGLT-2 inhibitors versus other glucose-lowering drugs in patients with type 2 diabetes: A real-world systematic review and meta-analysis

**DOI:** 10.1371/journal.pone.0244689

**Published:** 2021-02-19

**Authors:** Chun-xing Li, Shuo Liang, Lingyan Gao, Hua Liu

**Affiliations:** Department of Pharmacy, Aerospace Center Hospital, Beijing, China; Istituto Di Ricerche Farmacologiche Mario Negri, ITALY

## Abstract

**Background and aims:**

Glucose lowering agents that reduce the risk of major adverse cardiovascular events (MACE) would be considered a major advance. The reduction of cardiovascular risk by sodium-glucose cotransporter 2 inhibitors (SGLT-2i) has been confirmed by some large-scale randomized controlled studies (RCTs) and systematic reviews of RCTs, but exact indicators of cardiovascular risk remained controversial. Whether consistent results can be obtained in clinical practice is unclear. Therefore, in this meta-analysis, we analyzed the real-world effect of SGLT-2i on cardiovascular outcome in patients with type 2 diabetes mellitus (T2DM).

**Methods:**

We did a real-world systematic review and meta-analysis of cardiovascular outcome of SGLT-2i in patients with T2DM. We searched PubMed and Embase for trials published up to October 23, 2019. Data search and extraction were completed with a standardized data form and any discrepancies were resolved by consensus. The primary outcome was MACE and all-cause mortality (ACM). Secondary outcomes were hospitalization for heart failure (HHF), atrial fibrillation (AF), myocardial infarction (MI), stroke, cardiovascular mortality (CVM), unstable angina (UA), heart failure (HF). Odds ratio (OR) with 95% CIs were pooled across trials, and cardiovascular outcomes were stratified by baseline incidence of cardiovascular disease (CVD), usage rate of cardiovascular benefit drug, follow-up period and region.

**Results:**

Fourteen trials enrolling 3,157,259 patients were included. SGLT-2i reduced MACE (OR, 0.71; 95% CI 0.67,0.75, P<0.001) and ACM (OR, 0.53; 95% CI 0.49,0.57, P<0.001) compared to other glucose lowering drugs (oGLD). Compared with oGLD, SGLT-2i had significantly lowered the risk of HHF (OR, 0.56; 95% CI 0.46,0.68, P<0.001), MI (OR, 0.77; 95% CI 0.73,0.81, P<0.001), stroke (OR, 0.75; 95% CI 0.72,0.78, P<0.001), CVM (OR, 0.58; 95% CI 0.49,0.69, P<0.001) and HF (OR, 0.56; 95% CI 0.48,0.67, P<0.001), but there was no benefit from UA or AF. SGLT-2i significantly reduced the risk of severe hypoglycemia (OR, 0.78; 95% CI 0.69,0.90, P<0.001) and lower limb amputation (OR, 0.83; 95% CI 0.71,0.98, P<0.001), but it may increase the risk of diabetic ketoacidosis. Subgroup analysis showed SGLT-2i reduced the risk of MACE, ACM, HHF, MI, stroke, CVM and HF with a similar benefit regardless of the incidence of CVD was (20–30)% or < 15%, (15–30)% or <15% have been treated with GLP-1 receptor agonists (GLP-1RA), >80% or <70% have been treated with statins or both GLP-1RA and statins. SGLT-2i reduced the risk of ACM in low-risk population (P<0.001). No inconsistencies were found when stratification was performed at 1 or (3–4) years of follow-up except for BKA followed up for 1 year. SGLT-2i showed similar cardiovascular benefits in the Nordic countries, Asia and the United States.

**Conclusions:**

The predominant impact of SGLT-2i is on cardiovascular outcome driven predominantly by reduction in MACE, ACM, HHF, MI, stroke, CVM, HF, but not UA or AF. SGLT-2i has robust benefits on reducing MACE, ACM, HHF, MI, stroke, CVM and HF regardless of a history of usage rate of GLP-1RA and/or statins and /or metformin. SGLT-2i does not increase the risk of severe hypoglycemia and lower limb amputation.

## Introduction

Diabetes mellitus is a risk factor for cardiovascular disease (CVD) and has been associated with 2- to 4-fold higher mortality [1]. CVD remains a leading cause of morbidity and mortality in patients with type 1 or type 2 diabetes mellitus (T2DM) [2]. Since 2008, US Food and Drug Administration has mandated that all new antihyperglycaemic agents must be tested for cardiovascular safety in post-marketing endpoint trials [3]. Currently, there is a paradigm shift in T2DM management, moving from a primary objective of glucose control to a cardiovascular protection. There are many glucose-lowering drugs on the market, but several of them have demonstrated significant benefits of cardiovascular protection. Metformin [4, 5] and glucagon-like peptide 1 receptor agonists (GLP-1RA) are known to have cardiovascular protective effects [6–9]. Sodium glucose cotransporter 2 inhibitors (SGLT-2i) is a relatively new drug-class of glucose-lowering medications. The risks and benefits of SGLT-2i on cardiovascular outcomes have being studied in large prospective cardiovascular outcome trials (CVOTs): CANVAS (Canagliflozin) [10], DECLARE-TIMI 58 (Dapagliflozin) [11], EMPA-REG OUTCOME (Empagliflozin) [12], which have proven their efficacy to reduce major cardiovascular events (MACE) in patients with T2DM combined with cardiovascular disease. The cardiovascular protective effect of SGLT-2i was also confirmed in several meta-analysis and systematic evaluation of randomized controlled trial (RCT) [13–15]. However, whether these positive results could be extrapolated to patients in real world clinical practice is still unknown. SGLT-2i has been reported to be related to a possible increased risk of stroke. So, we pooled evidence from real-world studies to evaluate the cardiovascular benifits of these drugs.

## Materials and methods

### Study retrieval and selection

Our study protocol was registered in PROSPERO (CRD: 42019119236). Published observational studies and cohort studies on the cardiovascular outcomes of SGLT-2i in patients with T2DM were identified using PubMed and Embase databases. All eligible studies in English published until October 23, 2019 were included. The search strategy was consisted of a combination of the following Mesh terms and text words: sodium-dependent glucose co-transporter 2 inhibitors, sodium-glucose co-transporter 2 inhibitors, sodium/glucose cotransporter 2 inhibitors, sodium-glucose cotransporter 2 inhibitors, SGLT2 inhibitors, Ertugliflozin, Dapagliflozin, Canagliflozin, Empagliflozin, Ipragliflozin, Tofogliflozin, Luseogliflozin, sodium-glucose transporter 2 inhibitors, BMS-512148, cardiovascular disease [MeSH Terms], cardiovascular safety, CVD, major adverse cardiovascular event, major adverse cardiac events, MACE, cardiovascular outcomes, cardiovascular effects, cardiovascular risk factors, cardiovascular benefits, cardiovascular mortality, cardiovascular events, non-fatal myocardial infarction, myocardial infarction, rehospitalization, non-fatal stroke, hospitalization for heart failure, nonfatal myocardial infarction, nonfatal stroke, cardiovascular mortality, all-cause mortality, revascularization, cardiogenic death, stroke, vascular death, non-fatal acute myocardial infarction, hospitalization for unstable angina, heart failure requiring hospitalization. Meanwhile, randomized controlled trial, randomized control trial, and RCT were excluded.

### Criteria for inclusion

Studies that met the following conditions were included for this meta-analysis:

① Types of studies: prospective or retrospective observational studies, cohort studies.
② Study populations: participants were clearly diagnosed with T2DM, and participants were at least 18 years, no restrictions were applied in terms of sex or ethnicity.
③ Interventions: the experimental group administrated with SGLT-2i, the control group was treated with other glucose lowering drugs (oGLD).
④ Outcome measures: Primary outcomes: a composite cardiovascular endpoint: major adverse cardiovascular events (MACE), all-cause mortality (ACM); secondary outcomes: hospitalization for heart failure (HHF), atrial fibrillation (AF), myocardial infarction (MI), stroke, cardiovascular mortality (CVM), unstable angina (UA), heart failure (HF), severe hypoglycaemia, below the knee amputation (BKA). The list of title and abstract was assessed by 2 investigators to identify articles for full-text review. Any discrepancy or uncertainty was resolved by consensus or discussion with the other authors.

### Criteria for exclusion

① Participants <18 years.
② Randomized controlled study, review articles, case reports, letters to the editor.
③ Any other non-relevant studies were excluded from analysis.

### Data extraction

To avoid bias in the data abstraction process, a standardized extraction form was used, and the following data was extracted independently by 2 authors: first author, country, years of publication, study population, mean age of participants, number of patients, intervention plan, follow-up period, outcome measures, etc. Data extraction forms were cross-checked to verify accuracy and consistency of the extracted data. All data were checked by the third author and disagreements were resolved by discussion.

### Study quality assessment

The quality of the studies was independently assessed by 2 authors using the New castle-Ottawa Scale [16]. This scale rates studies on 3 major domains: selection, comparability, and exposure. A study can be awarded a maximum of 1 point for each numbered item within the selection and exposure categories, and a maximum of 2 points can be given for comparability. The full core is 9 points (highest quality), and we assigned scores of 0–3, 4–6, and 7–9 for low, moderate, and high quality of studies, respectively.

### Statistical analysis

The meta analysis was conducted by using Review Manager (version 5.3, The Cochrane Collaboration, Oxford, England). Stata 12.0 software (StataCorp, College Station, TX, United States) was used for publication bias analysis. Statistical heterogeneity between studies was assessed by the Cochran chi-square test complemented with the *I*^*2*^ statistic. If chi-square test was nonsignificant (*P*>0.10) and the *I*^2^ statistic was less than 50%, it indicated a lack of heterogeneity, and fixed effect model was adopted; on the contrary, the random effect model was used for analysis. Odds ratio (*OR*) was used to describe the classification variables. *OR* was estimated by the Mantel-Haenszel *χ*^*2*^ method, where *P* values < 0.05 were considered significantly different. Possible publication bias was assessed by Egger’s and Begg’s funnel plots, where *P* values < 0.05 indicated little publication bias.

## Results

### Description and quality evaluation of studies

**Fig 1:**
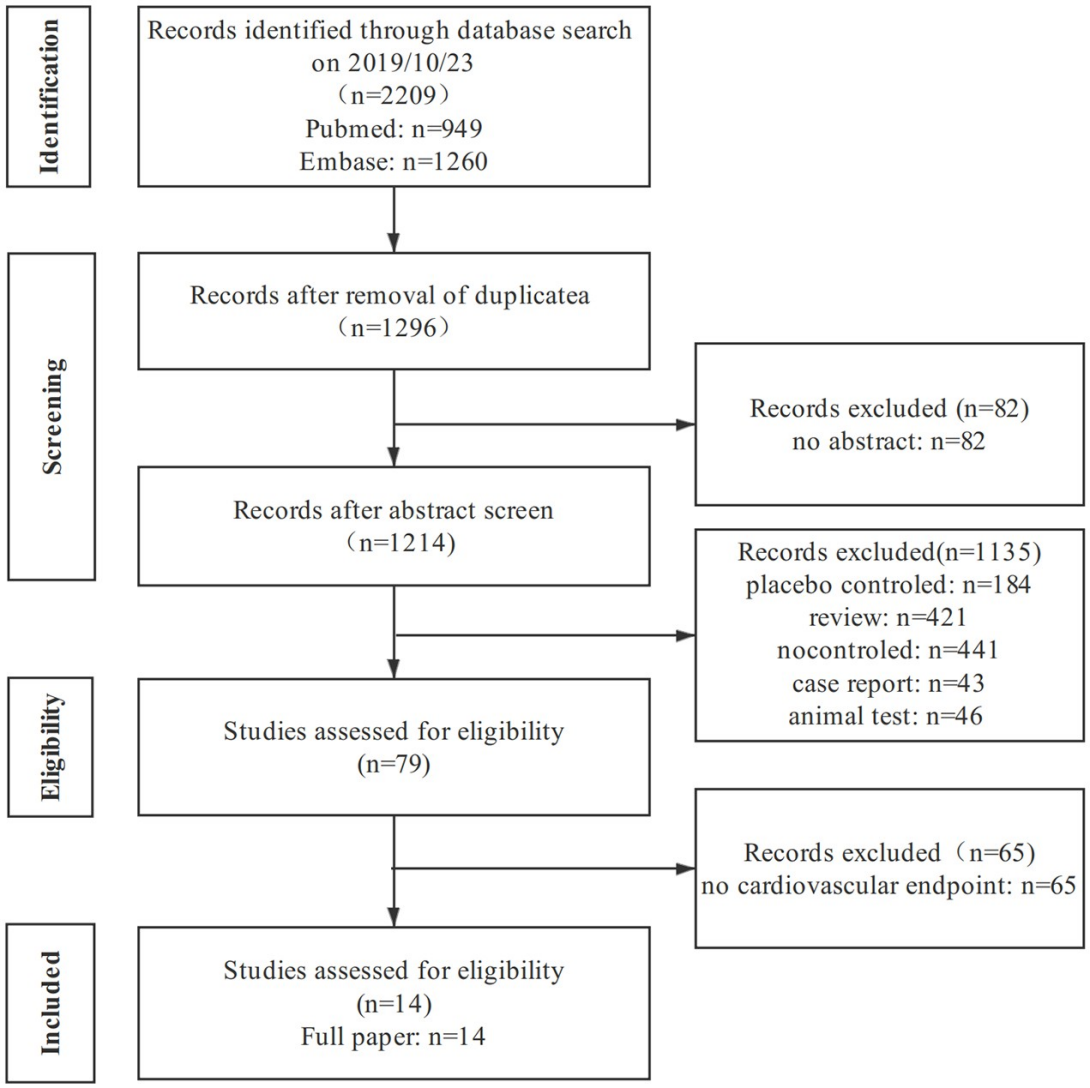
Screening process of included studies.

The literature screening process was shown in Fig 1. A total of 2209 studies were initially retrieved, and fourteen studies (3,157,259 patients) [17–30] were finally included after excluding those that did not meet the inclusion criteria. The characteristics of each study were presented in Table 1. In total, there were 1,127,629 enrolled participants in the trials who were treated with SGLT-2i, 2,029,630 were administrated with oGLD.

**Table 1:** Summary of included studies for systematic review and meta-analysis.

Study	Location	Design	Intervention	Patients (No.)	CVD (%)	Age (y)	Female (%)	Met	GLP-1RA	Statin	Duration of trial(y)
			Experiment/Control								
Persson F 2017 [17]	Denmark, Norway, Sweden	observational study	dapagliflozin/DPP4i	10227/30681	23.0/22.7	61.0±11.1/60.8±12.4	41.0/40.4	83.3/83.8	7.8/7.5	63.1/63.2	3.0
Tuolis KA 2017 [18]	United Kingdom	retrospective, open-cohort study	dapagliflozin/oGLD	4444/17680	<23.4/27.5	58.3±10.4 /58.5±10.4	41.4/41.4	-	-	88.4/84.7	0.78±0.54/0.74±0.53
										lipid-lowering drug	
Cahn A 2018 [19]	Israel	retrospective cohort study	SGLT-2i/DPP4i	6418/5604	33.4/27.5	62.3±9.4/64.2±12.1	38.3/43.0	-	28.2/2.3	-	0.5
Kosiborod M 2018 [20]	South Korea, Japan, Singapore, Israel, Australia, Canada	cohort study	SGLT-2i/oGLD	235064/235064	26.8/25.6	56.7±12.0/56.7±12.9	45.0/45.5	73.9/74.6	2.6/2.6	65.4/65.3	1.02/1.07
Birkeland KI 2017 [21]	Denmark, Norway, Sweden	observational study	SGLT-2i/oGLD	22830/68490	24.9/24.8	61.2±10.9/61.2±12.4	40.6/39.5	74.2/77.4	17.0/14.8	67.4/68.3	0.9±4.1
Udell JA 2018 [22]	US	population-based cohort study	SGLT-2i/ non-SGLT-2i	12629/12629	-	65.8±8.9/65.9±9.8	43.3/44.9	78.5/83.0	19.5/8.1	82.0/81.5	1.6
Dawwas GK 2018 [23]	US	retrospective cohort study	SGLT-2i/ sulfonylurea	62767/62767	11.9/11.2	54.0±12.4/54.0±9.6	47.7/47.6	57.8/58.8	13.9/12.5	-	1.0
			SGLT-2i/DPP4i	66633/66633	12.6/11.6	55.0±9.2/54±11.0	46.1/46.2	59.8/62.0	10.4/8.6	-	1.0
Kosiborod M 2017 [24]	United States, Germany, Sweden, Norway, Denmark, the United Kingdom	cohort study	SGLT-2i/oGLD	154528/154528	13.0/13.1	56.9±10.0/57.0±10.6	44.3/44.5	78.6/79.9	20.3/17.5	67.3/67.4	4.0
Cavender MA 2018 [25]	United States, United Kingdom, Sweden, Norway, Denmark	observational study	SGLT-2i/oGLD	19529/19764	100/100	62.7±9.7/63.5±10.4	35.9/36.6	75.2/79.6	21.8/19.6	81.2/82.0	1.02/1.07
			SGLT-2i/oGLD	133549/133314	0/0	56.0±9.8/56.0±10.5	45.5/45.7	79.2/80.0	20.1/17.3	65.6/65.5	1.02/1.07
Nyström T 2017 [26]	Sweden	observational study	dapagliflozin/ insulin	2047/4094	-	61.2±10.4/61.1±12.8	38.0/37.0	85.0/85.0	16.0/16.0	64.0/64.0	1.51/1.53
Pasternak B 2019 [27]	Denmark, Norway, Sweden	cohort study	SGLT-2i/DPP4i	20983/20983	-	61.0±10.0/61.0±10.0	40.0/40.0	79.0/79.0	10.0/10.0	67.0/67.0	1.1/1.7
										lipid-lowering drug	
Patorno E 2018 [28]	US	Retrospective cohort study	canagliflozin/DPP4i	17667/17667	-	56.5±10.6/56.5±10.7	44.9/45.0	15.7/15.4	5.9/5.9	60.2/60.3	0.6±0.5/0.6±0.5
			canagliflozin/ GLP-1RA	20539/20539	-	56.8±10.9/56.7±10.8	47.3/47.2	16.9/16.8	0/0	61.6/61.9	0.6±0.5/0.6±0.5
			canagliflozin/sulfonylurea	17354/17354	-	55.9±10.5/55.8±10.5	45.0/45.2	16.3/16.5	5.9/5.8	60.2/59.4	0.6±0.5/0.6±0.5
Ryan PB 2018 [29]	US	observational study	canagliflozin/all non-SGLT-2i	111332/445367	-	15.0–89.0/15.0–89.0	42.7–64.7/42.9–65.0	-	-	-	4.17
			canagliflozin/select non-SGLT-2i	120881/319976	-	15.0–89.0/15.0–89.0	42.7–64.7/42.9–65.0	-	-	-	4.17
			empagliflozin or dapagliflozin/all non-SGLT-2i	79626/350750	-	15.0–89.0/15.0–89.0	42.7–64.7/42.9–65.0	-	-	-	4.17
Norhammar A 2017 [30]	Norway, Sweden	cohort study	dapagliflozin/DPP4i	8582/25746	21.0/21.0	61.0/61.0	40.0/40.0	-	-	-	0.98

Met.:Metformin, CVD: Cardiovascular disease, SGLT-2i: SGLT-2 inhibitor, DPP4i: DPP-4 inhibitors, GLP-1RA: glucagon-like peptide (GLP)-1 receptor agonist, oGLD: other glucose-lowering drug.

The quality of the studies was assessed using the Newcastle-Ottawa Scale. Studies given greater than 4 stars were recognized as being moderate to high quality. The results of this assessment were shown in S1 Table. Of the 14 studies included, 12 had a score of 8, 1 had a score of 7 and 1 had a score of 6. The quality evaluations of all included literature were of moderate and high quality.

### Primary outcomes

#### MACE

**Fig 2:**
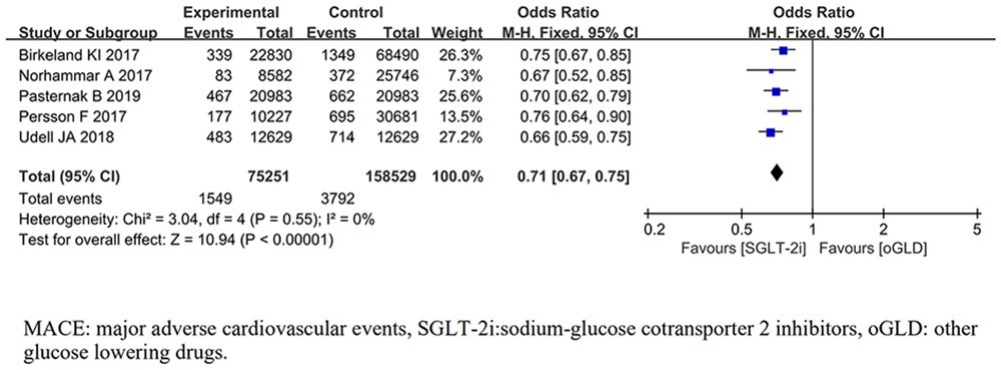
Forest plot of MACE for SGLT-2i and oGLD.

MACE was reported in 5 trials [17, 21, 22, 27, 30]. For the outcome of MACE, the pooled results from the fixed-effects model showed that compared with oGLD, SGLT-2i had significantly lowered the risk of MACE (*OR*, 0.71; 95% CI 0.67,0.75; *P*< 0.001;) (Fig 2). There was no heterogeneity across trials (*P* = 0.55; *I*^2^ = 0%).

#### All-cause mortality

**Fig 3:**
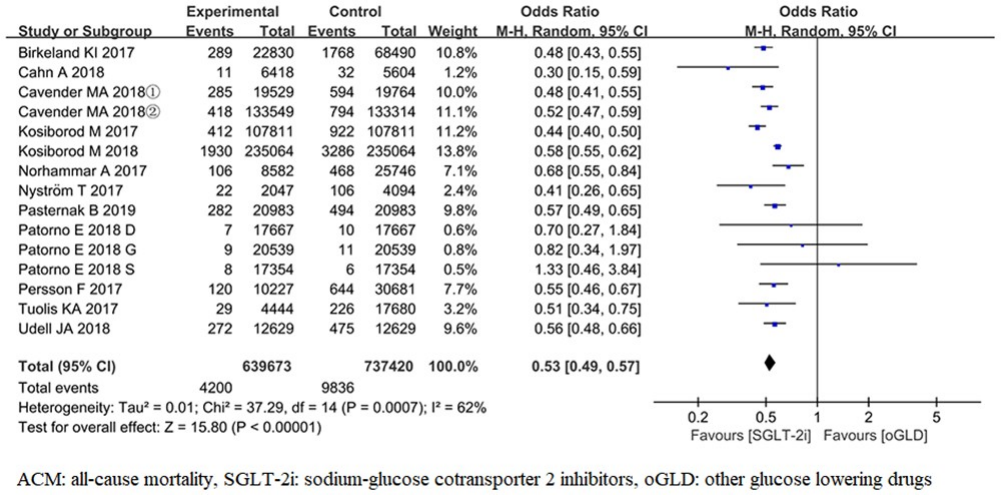
Forest plot of ACM for SGLT-2i and oGLD.

ACM was reported in 15 trials [17–22, 24–28, 30]. For the outcome of ACM, the pooled results from the random-effects model showed that compared with oGLD, SGLT-2i had significantly lowered the risk of ACM (*OR*, 0.53; 95% CI 0.49,0.57; *P*< 0.001) (Fig 3). There was heterogeneity across trials (*P*< 0.001; *I*^2^ = 62%).

### Secondary endpoint

#### CV outcomes

The pooled results of secondary endpoint were showed in Table 2, compared with oGLD, SGLT-2i had significantly lowered the risk of HHF, MI, stroke, CVM and HF (*P*< 0.001), but there was no benefit from UA (*P* = 0.51) or AF (*P* = 0.10).

**Table 2:** Results of meta-analysis on secondary endpoints.

Outcomes	Studies	Sample size	Heterogeneity	Model	*OR*	95% CI	*P*
HHF	12	2510050	*P*<0.001,*I*^2^ = 97%	Random	0.56	0.46,0.68	<0.001*
MI	10	1039500	*P* = 0.30,*I*^2^ = 15%	Fixed	0.77	0.73,0.81	<0.001*
Stroke	10	1039500	*P* = 0.23,*I*^2^ = 23%	Fixed	0.75	0.72,0.78	<0.001*
CVM	3	174194	*P* = 0.26,*I*^2^ = 26%	Fixed	0.58	0.49,0.69	<0.001*
UA	4	152028	*P* = 0.49,*I*^2^ = 0%	Fixed	0.92	0.73,1.17	0.51
HF	5	606922	*P*<0.001,*I*^2^ = 84%	Random	0.56	0.48,0.67	<0.001*
AF	2	132228	*P* = 0.77,*I*^2^ = 0%	Fixed	0.92	0.83,1.02	0.10
Severe hypoglycemia	3	138369	*P* = 0.29,*I*^2^ = 19%	Fixed	0.78	0.69,0.90	<0.001*
BKA	7	1718247	*P*<0.001,*I*^2^ = 75%	Random	0.83	0.71,0.98	0.02*

HHF: hospitalization for heart failure, MI: myocardial infarction, CVM: cardiovascular mortality, UA: unstable angina, HF: heart failure, AF: atrial fibrillation, BKA: below the knee amputation.

#### Safety outcomes

The pooled results of the incidence of severe hypoglycemia and BKA were showed in Table 2. Compared with oGLD, SGLT-2i had significantly lowered the risk of severe hypoglycemia (*P*< 0.001) and BKA (*P* = 0.02). The incidence rate of diabetic ketoacidosis (DKA) were 1.4 and 0.6 events per 1000 person years among SGLT-2i and DPP-4 inhibitors users, hazard ratios comparing SGLT-2i with DPP-4i was 2.14 (1.17–4.09).

### Subgroup analysis

#### Subgroup analysis according to the incidence of cardiovascular disease

In the included study, the incidence of CVD in T2DM patients at baseline was (11.2–33.4) %. *Toulis KA* [18] and *Cavender MA* [25] have divided people into low-risk and high-risk subset based on their cardiovascular risk. We performed a subgroup analysis based on the incidence of CVD, and they were divided into four groups: low-risk population, high risk population, population with incidence of CVD < 15% [23, 24] and population with incidence of CVD (20–30) % [17, 20, 21, 27, 30]. Table 3 showed the results of stratified analysis. SGLT-2i significantly decreased ACM compared with oGLD in low-risk population (*P*<0.001). SGLT-2i significantly decreased MACE, ACM, HHF, MI, stroke and CVM compared with oGLD whether the incidence of CVD was (20–30) % or < 15% (*P*<0.001). ACM in the high-risk population reported only in one trial, so the pooled results could not be obtained.

**Table 3:** The results of subgroup analysis based on the risk of cardiovascular disease.

Outcomes	Subgroup	Studies	Sample size	Heterogeneity	Model	*OR*	95% CI	*P*
MACE	20–30%	4	208522	*P* = 0.69,*I*^*2*^ = 0%	Fixed	0.73	0.67,0.78	<0.001*
ACM	20–30%	5	678650	*P* = 0.04,*I*^*2*^ = 59%	Random	0.56	0.51,0.62	<0.001*
	Low-risk	2	280219	*P* = 0.48,*I*^*2*^ = 0%	Fixed	0.52	0.46,0.58	<0.001*
HHF	20–30%	4	636684	*P* = 0.01,*I*^*2*^ = 73%	Random	0.69	0.60,0.79	<0.001*
MI	20–30%	4	644322	*P* = 0.57,*I*^*2*^ = 0%	Fixed	0.78	0.73,0.83	<0.001*
	<15%	2	258800	*P* = 0.25 *I*^*2*^ = 25%	Fixed	0.72	0.65,0.80	<0.001*
Stroke	20–30%	4	644322	*P* = 0.67,*I*^*2*^ = 0%	Fixed	0.76	0.73,0.80	<0.001*
	<15%	2	258800	*P* = 0.62,*I*^*2*^ = 0%	Fixed	0.68	0.62,0.75	<0.001*
CVM	20–30%	3	174194	*P* = 0.26,*I*^*2*^ = 26%	Fixed	0.58	0.49,0.69	<0.001*

MACE: major adverse cardiovascular events, ACM: all-cause mortality, HHF: hospitalization for heart failure, MI: myocardial infarction, CVM: cardiovascular mortality.

#### Subgroup analysis according to the usage rate of GLP-1RA, statins or metformin

In the included studies, patients have been previously given drugs with cardiovascular benefits. (15.4–85.0) % of patients have been treated with metformin, (5.0–28.2) % with GLP-1RA and (60.2–88.4) % with statins or lipid lowering drug.

Subgroup analysis was performed according to the different utilization rates of GLP-1RA and (or) statins (S2 Table.). In the population (15–30) % [19, 21, 22, 24–26] and <15% [17, 20, 27, 28] have been treated with GLP-1RA, SGLT-2i lowered the risk of MACE, ACM, HHF, MI, stroke and CVM more than oGLD with significantly statistical difference (*P*<0.05). In the population >80% [18, 22, 25]and <70% [17, 20, 21, 24–26, 28]have been treated with statins or lipid lowering drug, SGLT-2i lowered MACE, ACM, HHF, MI, stroke and CVM more than oGLD with significant statistical difference (*P*<0.001). In the population have been treated with statins (<70%) and GLP-1RA (<15%) at low rates [17, 20, 27, 28], SGLT-2i lowered the risk of MACE, ACM, HHF, MI, stroke and CVM with significant statistical difference (*P*<0.05). In the population have been treated with statins (>80%), GLP-1RA (15–30) % and metformin (>75%) at high rates [22, 25], SGLT-2i lowered ACM more than oGLD with significant statistical difference (*P*<0.001). There was no significant difference in UA between the two groups in the population have treated with GLP-1RA (<15%) and/or statins (<70%) (*P* = 0.51).

#### Subgroup analysis according to different regions

Subgroup analysis was performed according to studies in different regions, including the Nordic countries (Denmark, Norway, and Sweden) [17, 21, 25–27], Asia (Korea, Japan) [19, 20] and the United States [6, 7, 12, 13].

*HHF in Asia*. HHF in Asia was reported in 2 trials. the pooled results from the fixed-effects model showed that compared with oGLD, SGLT-2i had significantly lowered the risk of HHF (*OR*, 0.80; 95% CI 0.76,0.85; *P* <0.001) (S3 Table).

*In Nordic countries*. MACE, ACM, HHF, MI and stroke in the Nordic countries were reported respectively in 4, 5, 3, 3 and 3 trials. The pooled results showed that compared with oGLD, SGLT-2i had significantly lowered the risk of MACE, ACM, HHF, MI and stroke (*P* <0.05) (S3 Table).

*In the United States*. ACM, HHF, MI and stroke in the United States were reported respectively in 4, 7, 6 and 6 trials. The pooled results showed that compared with oGLD, SGLT-2i had significantly lowered the risk of ACM, HHF, MI and stroke (*P* <0.05) (S3 Table).

#### Subgroup analysis according to follow-up period

The subgroup was further analyzed according to the length of follow-up, The included studies were followed up for up to (3–4) years [17, 24, 29], 1 year [18, 20–23, 25–27, 30], and 6 months [19, 28]. Since the data of 6 months was only from two studies, we pooled the date of (3–4) years and 1 year. The results of subgroup analysis were consistent with the whole group analysis. SGLT-2i significantly reduced the risk of MACE, ACM, HHF, MI, stroke, CVM and HF than oGLD (*P*<0.05). There was no statistically significant difference in the risk of BKA in the SGLT-2i group after one-year follow-up (S4 Table).

### Sensitivity analyses

We deleted one single study from the overall pooled analysis each time to check the influence of the removed data set on the overall *OR*. If there is significant change, the results are considered unstable; otherwise, they are considered stable. The analysis results of all outcomes showed no significant change, so the research results were stable.

### Publication bias

All outcomes were examined by Egger’s and Begg’s, and the results (S5 Table) showed that publication bias might exist in MI (*P* = 0.049), while it did not exist in any other outcomes (*P*>0.05).

## Discussion

In this real-world meta-analysis of 14 trials enrolling 3,157,259 participants with T2DM, approximately (11.2~33.4) % of the study population had a previous CVD event (ischemic heart disease, stroke, and/or heart failure). The treatment regimen of the experimental group was SGLT-2i, including Dapagliflozin [17–27, 29], Ipraglifozin [19, 20], Canagliflozin [20–23, 25, 27–29], Empagliflozin [20–23, 25, 27, 29], Tofogliflozin [20], Luseogliflozin [20]. The control group was treated with oGLD, including DPP-4 inhibitors, metformin, sulfonylureas, GLP-1RA, thiazolidinediones, insulin. Both groups were combined with drugs that can provide cardiovascular benefits included angiotensin converting enzyme inhibitors, statins and antiplatelet. We found that compared with oGLD, SGLT-2i had been associated with significant risk reduction of MACE, ACM, HHF, MI, stroke, CVM and HF. However, we did not find that SGLT-2i resulted in a lower risk of UA and AF.

CANVAS trial showed patients treated with Canagliflozin had significantly lower risk of MACE, HHF than patients assigned to placebo, but ACM, CVM, MI, stroke were not considered to be significant [10]. DECLARE-TIMI 58 trial showed Dapagliflozin did not result in a lower rate of MACE, ACM, MI, CVM and stroke, but did result in a lower rate of HHF [11]. EMPA-REG OUTCOME trial showed Empagliflozin reduced the risk of MACE, ACM, HHF, CVM, while, there were no significant between-group differences in the occurrence of MI or stroke [12]. Participants in CANVAS trial, DECLARE–TIMI 58 trial and EMPA-REG OUTCOME trial were diabetic patients with established cardiovascular disease or at high risk for cardiovascular disease. In our pooled analysis, only about (10–30) % of patients were diagnosed with CVD, with a lower cardiovascular prevalence than in those three randomized controlled trials (RCTs). But our meta-analysis from the real world showed that SGLT-2i was associated with greater cardiovascular benefit than the three RCTs that have been published. Strict inclusion and exclusion criteria and rigorous safety monitoring may limit the generalizability of RCT results.

Our results were consistent with the results of the meta-analysis from the RCT of Usman MS, et al. The difference was that Usman MS’s study showed no significant difference in stroke [31]. Zelniker TA, et al found SGLT-2i reduced the risk of MACE by 11% with benefit only seen in patients with atherosclerotic cardiovascular disease and not in those without [15]. We found different results from subgroup analyses based on the prevalence of CVD. SGLT-2i significantly decreased the risk of MACE, ACM, HHF, MI, stroke and CVM compared with oGLD whether the incidence of CVD was (20–30) % or < 15%. SGLT-2i significantly decreased ACM compared with oGLD in low-risk population, which only came from a pooled analysis of two studies. Cohort studies showed Canagliflozin had no treatment heterogeneity between patients with and without established heart failure or CVD [28]. Unfortunately, we could not do more than ACM in low risk populations. Therefore, more studies are needed to verify the cardiovascular benefits of SGLT-2i in people with low risk of CVD.

The usage rate of cardiovascular benefit drugs such as GLP-1RA, Metformin, statins in the included studies varied. When stratified according to the usage rate of drugs for cardiovascular benefit at baseline, we got consistent results regardless of high or low usage rate. SGLT-2i lowered MACE, ACM, HHF, MI, stroke and CVM more than oGLD with significantly statistical difference. Although the studies included were real-world cohort studies, they were generally balanced by propensity score matching before data analysis. There was no significant difference in the use of other drugs between SGLT-2i group and oGLD group, which suggested that the cardiovascular benefits were mainly due to the use of SGLT-2i.

The participants included came from different regions such as the Nordic countries, the United States, Asia and the Middle East. Different regions have different insurance types or no insurance coverage, as commercially insured patients are more likely to have differential socioeconomic status, drug adherence, and risk factors for cardiovascular disease. We performed stratified analysis on the population from different regions. No matter in the Nordic countries or the United States, SGLT-2i can significantly reduced the risk of ACM, HHF, MI and stroke. Unfortunately, studies of participants from Asia only had two studies, pooled analysis of two Asia studies showed SGLT-2i can significantly reduce the risk of HHF in Asia.

The follow-up time of the included study was (0.6–4.0) years, Insufficient follow-up time may affect the results of the study. Subgroup analysis showed SGLT-2i significantly reduced MACE, ACM, HHF, MI, stroke, CVM and HF than oGLD whether they were followed up for (3–4) years or 1 year. SGLT-2i failed to significantly reduce ACM in the pooled two studies with a follow-up of 6 months, which depended largely on Patorno E’s study [28]. Interventions in one of the cohort was SGLT-2i *vs*. GLP-1RA of Patorno E’s study [28]. A network meta-analysis showed the use of SGLT-2i or GLP-1RA was associated with similar lower mortality than DPP-4 inhibitors or placebo or no treatment [32]. On the other hand, the short duration of follow-up explained the lower observed death rates compared with the long duration trials.

In terms of safety, our study showed that SGLT-2i reduces the risk of severe hypoglycemia and lower limb amputation. Burt, there was no statistically significant difference in the risk of lower limb amputation in the SGLT-2i group after one-year follow-up. Currently, studies on the risk of lower limb amputation are controversial. Several observational studies have shown that SGLT-2i does not increase the risk of lower limb amputation [33–35]. But other observational studies have shown contrary results [36, 37]. Several meta analyses from RCT all showed that SGLT-2i were not significantly associated with risk of amputation [38, 39]. Subgroup analysis showed an increased incidence of amputation in participants using Canagliflozin [38]. Another meta analysis showed that neither Canagliflozin nor SGLT-2i increase the risk of amputation [40].

Only one of the included studies mentioned that SGLT-2i increased the risk of DKA. Register based cohort study showed SGLT-2i was associated with an increased risk of DKA (HR: 2.14, 1.01–4.52) compared with GLP-1RA [36]. A multicenter cohort study found SGLT-2i was associated with an increased risk for DKA (HR:2.85, 1.99–4.08) compared with DPP-4 inhibitors [41]. A meta-analysis of random controlled trials showed that SGLT-2i did not increase the risk of DKA (MH-OR:1.14, 0.45–2.88; *P* = 0.78) [42]. In summary, real world study data showed that SGLT-2i increases the risk of DKA differently than RCT data.

Our study included a large sample size and performed a subgroup analysis. However, our study also had several limitations as following: Firstly, We used aggregated study-level data rather than individual participant data. Secondly, fewer included studies performed subgroup analysis on the high-risk and low-risk groups of CVD. Therefore, besides SGLT-2i could reduce the risk of ACM, we could not find out more about the cardiovascular benefits of SGLT-2i in the low-risk population. Thirdly, although stratified analyses were performed based on the prevalence of CVD and the use of drugs for cardiovascular benefits in the included studies, approximately 40% of the included studies were not included in the subgroup analysis because no detailed cardiovascular prevalence or use of drugs for cardiovascular benefits were reported. Fourthly, participants in the study were mainly from Nordic countries and the United States, only a small quantity of them were from Asia. Therefore, we need cardiovascular outcomes of SGLT-2i for participants in East Asia especially China. Fifthly, there were few studies on severe adverse reactions, so whether SGLT-2i increases the risk of BKA and DKA cannot be fully assessed. Sixthly, unfortunately, we were unable to conduct a pooled analysis of the effects of SGLT-2i on blood pressure due to the lack of relevant data in the included studies. Finally, some heterogeneity appears to exist between different brands of SGLT-2i, due to the limited data we cannot fully explore through subgroup.

## Conclusion

In summary, our study showed that among patients with T2DM who had an increased risk of CVD, SGLT-2i significantly reduced the risk for MACE, ACM, HHF, MI, stroke, CVM and HF regardless of the incidence of CVD was (20–30) % or <15%, (15–30) % or <15% have been treated with GLP-1RA, >80% or <70% have been treated with statins or both GLP-1RA and statins. No inconsistencies were found when stratification was performed at 1 and (3–4) years of follow-up. SGLT-2i showed similar cardiovascular benefits in Nordic countries, Asia and the United States. In terms of severe adverse reactions, our study showed that SGLT-2i did not increase the risk of severe hypoglycemia and lower limb amputation, but whether SGLT-2i increases the risk of lower limb amputation still needs further evaluation. Observational studies have generally shown that SGLT-2i increased the risk of diabetic ketosis. Therefore, clinician should pay attention to monitoring adverse reactions in diabetes treatments.

## Supporting information

S1 Table Quality assessment of included studies according to the Newcastle-Ottawa Scale. (DOCX)

S2 Table The results of subgroup analysis based on the usage rate of GLP-1RA and/or statins. (DOCX)

S3 Table Cardiovascular outcomes of subgroup analysis according to different regions. (DOCX)

S4 Table Cardiovascular outcomes of subgroup analysis according to follow-up period. (DOCX)

S5 Table The publication bias of Begg’s test and Egger’s test. (DOCX)

S1 Checklist PRISMA 2009 checklist. (DOC)

S1 Data (XLSX)

## Abbreviations

ACM: all-cause mortality
AF: atrial fibrillation
BKA: below the knee amputation
CIs: confidence intervals
CVD: cardiovascular disease
CVM: cardiovascular mortality
CVOTs: cardiovascular outcome trials
DKA: Diabetic ketoacidosis
GLP-1RA: GLP-1 receptor agonists
HF: heart failure
HHF: hospitalization for heart failure
MACE: major adverse cardiovascular events
MI: myocardial infarction
oGLD: other glucose lowering drugs
OR: Odds ratio
RCT: randomized controlled trial
SGLT-2i: sodium-glucose cotransporter 2 inhibitors
T2DM: type 2 diabetes mellitus
UA: unstable angina
