# Supplementary material for: Cardiovascular outcomes associated with SGLT-2 inhibitors versus other glucose-lowering drugs in patients with type 2 diabetes: A real-world systematic review and meta-analysis
Source: PLoS One. 2021 Feb 19;16(2):e0244689. doi: 10.1371/journal.pone.0244689 (PMC7895346; doi:10.1371/journal.pone.0244689)
Supplement: S1 Table — (DOCX) [file pone.0244689.s001.docx]

**S1 Table. Quality assessment of included studies according to the Newcastle-Ottawa Scale.**

| **Study** | Selection | | | | **Comparability** | | **Outcome** | | | **In total** |
| --- | --- | --- | --- | --- | --- | --- | --- | --- | --- | --- |
|  | 1^*^ | 2^*^ | 3^*^ | 4^*^ | 5^*^ | 6^*^ | 7^*^ | 8^*^ | 9^*^ |  |
| Persson F 2017^[17]^ | 1 | 1 | 1 | 1 | 1 | 0 | 1 | 1 | 1 | 8 |
| Tuolis KA 2017^[18]^ | 1 | 1 | 1 | 1 | 1 | 0 | 1 | 1 | 1 | 8 |
| Cahn A 2018^[19]^ | 1 | 1 | 1 | 1 | 0 | 0 | 1 | 0 | 1 | 6 |
| Kosiborod M 2018^[20]^ | 1 | 1 | 1 | 1 | 1 | 0 | 1 | 1 | 1 | 8 |
| Birkeland KI 2017^[21]^ | 1 | 1 | 1 | 1 | 1 | 0 | 1 | 1 | 1 | 8 |
| Udell JA 2018^[22]^ | 1 | 1 | 1 | 1 | 1 | 0 | 1 | 1 | 1 | 8 |
| Dawwas GK 2018^[23]^ | 1 | 1 | 1 | 1 | 1 | 0 | 1 | 1 | 1 | 8 |
| Kosiborod M 2017^[24]^ | 1 | 1 | 1 | 1 | 1 | 0 | 1 | 1 | 1 | 8 |
| Cavender MA 2018^[25]^ | 1 | 1 | 1 | 1 | 1 | 0 | 1 | 1 | 1 | 8 |
| Nyström T 2017^[26]^ | 1 | 1 | 1 | 1 | 1 | 0 | 1 | 1 | 1 | 8 |
| Pasternak B 2019^[27]^ | 1 | 1 | 1 | 1 | 1 | 0 | 1 | 1 | 1 | 8 |
| Patorno E 2018^[28]^ | 1 | 1 | 1 | 1 | 1 | 0 | 1 | 0 | 1 | 7 |
| Ryan PB 2018^[29]^ | 1 | 1 | 1 | 1 | 1 | 0 | 1 | 1 | 1 | 8 |
| Norhammar A 2017^[30]^ | 1 | 1 | 1 | 1 | 1 | 0 | 1 | 1 | 1 | 8 |

1^*^: Representativeness of the exposed cohort, 2^*^: Selection of the non exposed cohort, 3^*^: Ascertainment of exposure, 4^*^: Demonstration that outcome of interest was not present at start of study, 5^*^: Study controls for the most important factor, 6^*^: Study controls for any additional factor, 7^*^: Assessment of outcome, 8^*^: Was follow-up long enough for outcomes to occur, 9^*^: Adequacy of follow up of cohorts.
