# Supplementary material for: Cardiovascular outcomes associated with SGLT-2 inhibitors versus other glucose-lowering drugs in patients with type 2 diabetes: A real-world systematic review and meta-analysis
Source: PLoS One. 2021 Feb 19;16(2):e0244689. doi: 10.1371/journal.pone.0244689 (PMC7895346; doi:10.1371/journal.pone.0244689)
Supplement: S2 Table — (DOCX) [file pone.0244689.s002.docx]

**S2 Table. The results of subgroup analysis based on the usage rate of GLP-1RA and/or statins**

| **Outcomes** | **Subgroup** | **Studies** | **Sample size** | **Heterogeneity** | **Model** | ***OR*** | **95% CI** | ***P*** |
| --- | --- | --- | --- | --- | --- | --- | --- | --- |
| MACE | 15-30% (GLP-1RA) | 2 | 116578 | *P=*0.15,*I*^2^=51% | Random | 0.71 | 0.63,0.80 | <0.001^*^ |
|  | <15% (GLP-1RA) | 2 | 82874 | *P=*0.42,*I*^2^=0% | Fixed | 0.72 | 0.65,0.79 | <0.001^*^ |
|  | <70% (Statin) | 3 | 174194 | *P=*0.63,*I*^2^=0% | Fixed | 0.73 | 0.68,0.79 | <0.001^*^ |
|  | <70% (Statin)，  <15% (GLP-1RA) | 2 | 82874 | *P=*0.42,*I*^2^=0% | Fixed | 0.72 | 0.65,0.79 | <0.001^*^ |
| ACM | 15-30% (GLP-1RA) | 7 | 656519 | *P*=0.13,*I*^2^=39% | Fixed | 0.49 | 0.46,0.52 | <0.001^*^ |
|  | <15% (GLP-1RA) | 6 | 664122 | *P*=0.63,*I*^2^=0% | Fixed | 0.58 | 0.55,0.61 | <0.001^*^ |
|  | >80% (Statin) | 3 | 86675 | *P*=0.30,*I*^2^=17% | Fixed | 0.52 | 0.47,0.57 | <0.001^*^ |
|  | <70% (Statin) | 10 | 1244068 | *P*=0.001,*I*^2^=67% | Random | 0.53 | 0.48,0.58 | <0.001^*^ |
|  | >80%(Statin),>75%(Met) 15-30% (GLP-1RA) | 2 | 64551 | *P*=0.12,*I*^2^=58% | Fixed | 0.52 | 0.47,0.57 | <0.001^*^ |
|  | <70% (Statin)，  <15% (GLP-1RA) | 6 | 664122 | *P*=0.63,*I*^2^=0% | Fixed | 0.58 | 0.55,0.61 | <0.001^*^ |
| HHF | 15-30% (GLP-1RA) | 3 | 425634 | *P=*0.41,*I*^2^=0% | Fixed | 0.63 | 0.58,0.69 | <0.001^*^ |
|  | <15% (GLP-1RA) | 5 | 622156 | *P=*0.002,*I*^2^=76% | Random | 0.66 | 0.55,0.79 | <0.001^*^ |
|  | <70% (Statin) | 7 | 1022532 | *P<*0.001,*I*^2^=78% | Random | 0.66 | 0.58,0.75 | <0.001^*^ |
|  | <70% (Statin)，  <15% (GLP-1RA) | 5 | 622156 | *P=*0.002,*I*^2^=76% | Random | 0.66 | 0.55,0.79 | <0.001^*^ |
| MI | 15-30% (GLP-1RA) | 2 | 116578 | *P*=0.85,*I*^2^=0% | Fixed | 0.83 | 0.75,0.96 | 0.01^*^ |
|  | <15% (GLP-1RA) | 8 | 922922 | *P*=0.23,*I*^2^=25% | Fixed | 0.76 | 0.72,0.81 | <0.001^*^ |
|  | <70% (Statin) | 7 | 755442 | *P*=0.32,*I*^2^=10% | Fixed | 0.79 | 0.74,0.84 | <0.001^*^ |
|  | <70% (Statin)，  <15% (GLP-1RA) | 6 | 664122 | *P*=0.29,*I*^2^=19% | Fixed | 0.78 | 0.73,0.84 | <0.001^*^ |
| Stroke | 15-30% (GLP-1RA) | 2 | 116578 | *P*=0.87,*I*^2^=0% | Fixed | 0.85 | 0.73,0.98 | 0.03^*^ |
|  | <15% (GLP-1RA) | 8 | 922922 | *P*=0.24,*I*^2^=24% | Fixed | 0.75 | 0.72,0.78 | <0.001^*^ |
|  | <70% (Statin) | 7 | 755442 | *P*=0.53,*I*^2^=0% | Fixed | 0.77 | 0.74,0.80 | <0.001^*^ |
|  | <70% (Statin)，  <15% (GLP-1RA) | 6 | 664122 | *P*=0.53,*I*^2^=0% | Fixed | 0.76 | 0.73,0.80 | <0.001^*^ |
| CVM | <15% (GLP-1RA) | 2 | 82874 | *P*=0.49,*I*^2^=0% | Fixed | 0.64 | 0.53,0.79 | <0.001^*^ |
|  | <70% (Statin) | 3 | 174194 | *P=*0.26*,I^2^*=26% | Fixed | 0.58 | 0.49,0.69 | <0.001^*^ |
|  | <70% (Statin)，  <15% (GLP-1RA) | 2 | 82874 | *P*=0.49,*I*^2^=0% | Fixed | 0.64 | 0.53,0.79 | <0.001^*^ |
| UA | <15% (GLP-1RA) | 4 | 152028 | P=0.49,I^2^=0% | Fixed | 0.92 | 0.73,1.17 | 0.51 |
|  | <70% (Statin) | 4 | 152028 | P=0.49,I^2^=0% | Fixed | 0.92 | 0.73,1.17 | 0.51 |
|  | <70% (Statin)，  <15% (GLP-1RA) | 4 | 152028 | P=0.49,I^2^=0% | Fixed | 0.92 | 0.73,1.17 | 0.51 |
| HF | 15-30% (GLP-1RA) | 2 | 306156 | *P*=0.10,*I*^2^=64% | Random | 0.67 | 0.55,0.82 | <0.001^*^ |
|  | <15% (GLP-1RA) | 3 | 300766 | *P*=0.25,*I*^2^=27% | Fixed | 0.51 | 0.47,0.55 | <0.001^*^ |
|  | <70% (Statin) | 2 | 308829 | *P*=0.17,*I*^2^=48% | Fixed | 0.54 | 0.47,0.63 | <0.001^*^ |

MACE: major adverse cardiovascular events, ACM: all-cause mortality, HHF: hospitalization for heart failure, MI: myocardial infarction, CVM: cardiovascular mortality, UA: unstable angina, HF: heart failure, AF: atrial fibrillation, GLP-1RA: Glucagon-like peptide 1 receptor agonist, Met：metformin, OR: odds ratio.
