# Supplementary material for: Cardiovascular outcomes associated with SGLT-2 inhibitors versus other glucose-lowering drugs in patients with type 2 diabetes: A real-world systematic review and meta-analysis
Source: PLoS One. 2021 Feb 19;16(2):e0244689. doi: 10.1371/journal.pone.0244689 (PMC7895346; doi:10.1371/journal.pone.0244689)
Supplement: S3 Table — (DOCX) [file pone.0244689.s003.docx]

**S3 Table. Cardiovascular outcomes of subgroup analysis according to different regions**

| **Outcomes** | **Subgroup** | **Studies** | **Sample size** | **Heterogeneity** | **Model** | ***OR*** | **95% CI** | ***P*** |
| --- | --- | --- | --- | --- | --- | --- | --- | --- |
| MACE | Nordic | 4 | 208522 | *P=*0.69,*I*^2^=0% | Random | 0.73 | 0.67,0.78 | <0.001^*^ |
| ACM | Nordic | 5 | 214663 | *P*=0.06,*I*^2^=56% | Random | 0.55 | 0.48,0.62 | <0.001^*^ |
|  | US | 4 | 136378 | *P*=0.35,*I*^2^=8% | Fixed | 0.58 | 0.50,0.67 | <0.001^*^ |
| HHF | Nordic | 3 | 166556 | *P*=0.62,*I*^2^=0% | Fixed | 0.65 | 0.58,0.72 | <0.001^*^ |
|  | US | 7 | 1564310 | *P*<0.001,*I*^2^=91% | Random | 0.49 | 0.41,0.58 | <0.001^*^ |
|  | Asia | 2 | 404424 | *P*=0.36,*I*^2^=0% | Fixed | 0.80 | 0.76,0.85 | <0.001^*^ |
| MI | Nordic | 3 | 174194 | *P*=0.46,*I*^2^=0% | Fixed | 0.80 | 0.72,0.89 | <0.001^*^ |
|  | US | 6 | 395178 | *P*=0.13,*I*^2^=42% | Fixed | 0.77 | 0.71,0.84 | <0.001^*^ |
| Stroke | Nordic | 3 | 174194 | *P*=0.47,*I*^2^=0% | Fixed | 0.77 | 0.69, 0.87 | <0.001^*^ |
|  | US | 6 | 395178 | *P*=0.12,*I*^2^=43% | Fixed | 0.72 | 0.67,0.78 | <0.001^*^ |

MACE: major adverse cardiovascular events, ACM: all-cause mortality, HHF: hospitalization for heart failure, MI: myocardial infarction, OR: odds ratio.
