# Supplementary material for: Cardiovascular outcomes associated with SGLT-2 inhibitors versus other glucose-lowering drugs in patients with type 2 diabetes: A real-world systematic review and meta-analysis
Source: PLoS One. 2021 Feb 19;16(2):e0244689. doi: 10.1371/journal.pone.0244689 (PMC7895346; doi:10.1371/journal.pone.0244689)
Supplement: S4 Table — (DOCX) [file pone.0244689.s004.docx]

**S4 Table. Cardiovascular outcomes of subgroup analysis according to follow-up period**

| **Outcomes** | **Subgroup** | **Studies** | **Sample size** | **Heterogeneity** | **Model** | ***OR*** | **95% CI** | ***P*** |
| --- | --- | --- | --- | --- | --- | --- | --- | --- |
| MACE | 1Y | 4 | 191814 | *P*=0.4,*I*^2^=0% | Fixed | 0.71 | 0.66,0.77 | <0.001^*^ |
| ACM | 3-4Y | 2 | 256530 | *P*=0.06,*I*^2^=72% | Random | 0.49 | 0.40,0.61 | <0.001^*^ |
|  | 1Y | 8 | 997421 | *P*=0.02,*I*^2^=56% | Random | 0.54 | 0.50,0.58 | <0.001^*^ |
|  | 0.6Y | 4 | 123142 | *P*=0.08,*I*^2^=55% | Random | 0.64 | 0.33,1.25 | 0.19 |
| HHF | 3-4Y | 5 | 1777896 | *P<*0.001,*I*^2^=94% | Random | 0.47 | 0.38,0.57 | <0.001^*^ |
|  | 1Y | 4 | 621034 | *P=*0.006,*I*^2^=76% | Random | 0.68 | 0.58,0.79 | <0.001^*^ |
| MI | 1Y | 6 | 887472 | *P*=0.54,*I*^2^=0% | Fixed | 0.76 | 0.72,0.80 | <0.001^*^ |
| Stroke | 1Y | 6 | 887472 | *P*=0.17,*I*^2^=35% | Fixed | 0.75 | 0.72,0.78 | <0.001^*^ |
| CVM | 1Y | 2 | 133286 | *P*=0.26,*I*^2^=21% | Fixed | 0.55 | 0.46,0.66 | <0.001^*^ |
| HF | 1Y | 5 | 606922 | *P<*0.001,*I^2^=*84% | Random | 0.56 | 0.48,0.67 | <0.001^*^ |
| BKA | 1Y | 4 | 317688 | *P*=0.001,*I*^2^=81% | Random | 0.86 | 0.59,1.25 | 0.43 |

MACE: major adverse cardiovascular events, ACM: all-cause mortality, HHF: hospitalization for heart failure, MI: myocardial infarction, CVM: cardiovascular mortality, HF: heart failure, BKA: below the knee amputation, Y:year, OR: odds ratio.
