# Supplementary material for: Cardiovascular outcomes associated with SGLT-2 inhibitors versus other glucose-lowering drugs in patients with type 2 diabetes: A real-world systematic review and meta-analysis
Source: PLoS One. 2021 Feb 19;16(2):e0244689. doi: 10.1371/journal.pone.0244689 (PMC7895346; doi:10.1371/journal.pone.0244689)
Supplement: S5 Table — (DOCX) [file pone.0244689.s005.docx]

**S5 Table. The Publication bias of Begg's Test and Egger's test**

| Outcomes | P | |
| --- | --- | --- |
|  | Begg's Test | Egger's test |
| MACE | 0.086 | 0.050 |
| ACM | 0.592 | 0.830 |
| HHF | 0.876 | 0.581 |
| MI | 0.049 | 0.029 |
| Stroke | 0.283 | 0.612 |
| CVM | 1.000 | 0.680 |
| UA | 0.734 | 0.715 |
| HF | 0.806 | 0.852 |
| Severe hypoglycemia | 1.000 | 0.986 |
| BKA | 0.764 | 0.675 |

MACE: major adverse cardiovascular events, ACM: all-cause mortality, HHF: hospitalization for heart failure, MI: myocardial infarction, CVM: cardiovascular mortality, UA: unstable angina, HF: heart failure, AF: atrial fibrillation, BKA: below the knee amputation.
